# Chemotherapy induces Notch1-dependent MRP1 up-regulation, inhibition of which sensitizes breast cancer cells to chemotherapy

**DOI:** 10.1186/s12885-015-1625-y

**Published:** 2015-09-11

**Authors:** Baek Kim, Sam L. Stephen, Andrew M. Hanby, Kieran Horgan, Sarah L. Perry, Julie Richardson, Elizabeth A. Roundhill, Elizabeth M. A. Valleley, Eldo T. Verghese, Bethany J. Williams, James L. Thorne, Thomas A. Hughes

**Affiliations:** 1 School of Medicine, University of Leeds, Leeds, UK; 2 Department of Breast Surgery, Leeds Teaching Hospitals NHS Trust, Leeds, UK; 3 Department of Histopathology, Leeds Teaching Hospitals NHS Trust, Leeds, UK; 4 School of Food Science and Nutrition, University of Leeds, Leeds, UK

## Abstract

**Background:**

Multi-drug Resistance associated Protein-1 (MRP1) can export chemotherapeutics from cancer cells and is implicated in chemoresistance, particularly as is it known to be up-regulated by chemotherapeutics. Our aims in this study were to determine whether activation of Notch signalling is responsible for chemotherapy-induced MRP1 expression Notch in breast cancers, and whether this pathway can be manipulated with an inhibitor of Notch activity.

**Methods:**

MRP1 and Notch1 were investigated in 29 patients treated with neoadjuvant chemotherapy (NAC) for breast cancer, using immunohistochemistry on matched biopsy (pre-NAC) and surgical samples (post-NAC). Breast epithelial cell cultures (T47D, HB2) were treated with doxorubicin in the presence and absence of functional Notch1, and qPCR, siRNA, Western blots, ELISAs and flow-cytometry were used to establish interactions.

**Results:**

In clinical samples, Notch1 was activated by neoadjuvant chemotherapy (Wilcoxon signed-rank *p* < 0.0001) and this correlated with induction of MRP1 expression (rho = 0.6 *p* = 0.0008). In breast cell lines, doxorubicin induced MRP1 expression and function (non-linear regression *p* < 0.004). In the breast cancer line T47D, doxorubicin activated Notch1 and, critically, inhibition of Notch1 activation with the γ-secretase inhibitor DAPT abolished the doxorubicin-induced increase in MRP1 expression and function (*t*-test *p* < 0.05), resulting in enhanced cellular retention of doxorubicin and increased doxorubicin-induced apoptosis (*t*-test *p* = 0.0002). In HB2 cells, an immortal but non-cancer derived breast cell line, Notch1-independent MRP1 induction was noted and DAPT did not enhance doxorubicin-induced apoptosis.

**Conclusions:**

Notch inhibitors may have potential in sensitizing breast cancer cells to chemotherapeutics and therefore in tackling chemoresistance.

**Electronic supplementary material:**

The online version of this article (doi:10.1186/s12885-015-1625-y) contains supplementary material, which is available to authorized users.

## Background

Therapy for primary breast cancer usually involves tumour resection combined with radiotherapy, endocrine-therapy, and/or chemotherapy. Approximately 30 % of patients receive chemotherapy, typically including anthracyclines and/or taxanes [1]. However, resistance to chemotherapy is a substantial problem, reflected in subsequent presentations with recurrences. An important research goal in breast cancer is to understand better the molecular mechanisms that lead to failure of chemotherapy treatment regimens [2]. ATP-binding cassette (ABC) proteins are a family of xenobiotic drug transporters many of which are capable of exporting chemotherapeutic drugs from cells. Accordingly, some ABC proteins have been implicated in chemotherapy resistance. For example, Multi-drug Resistance associated Protein-1 (MRP1; encoded by the gene *ABCC1*) can export the chemotherapeutics epirubicin and doxorubicin [3, 4], and is overexpressed in a variety of multi-drug resistant cancer cell lines [5, 6]. Furthermore, MRP1 expression has been linked with poor clinical outcomes in breast cancer [7–9], although this correlation has not been observed consistently in all such studies [10], perhaps due to lack of statistical power. Various small molecule inhibitors of ABCs have been developed as potential sensitizing agents for chemotherapy, yet these have not progressed from trials to clinic largely because they are not well tolerated in normal cells, particularly of the liver, kidneys and intestine [11]. Elucidation and targeting of tumour specific pathways that regulate expression of ABCs may present methods to inhibit ABC function in cancer cells, thereby sensitizing them to chemotherapy agents, while reducing side effects associated with unwanted influences in normal cells.

The work described here has linked two independent published studies to suggest a potential cancer-specific regulatory pathway for MRP1. First, MRP1 was shown to be a direct transcriptional target of Notch1 in the multi-drug resistant breast cancer cell line, MCF7-VP [12]. Secondly, Notch signalling was shown to be significantly up-regulated in breast tumours of patients treated with neoadjuvant chemotherapy (NAC) [13]. Based on these observations, our hypothesis was that in some breast cancers chemotherapy drives MRP1-dependent acquired resistance via Notch signalling.

Notch molecules are a family of four (Notch1 to 4) signalling molecules that can act as transcriptional co-regulators; Notch1 is implicated in mammary gland development [14], breast cancer [15–17] and chemoresistance to doxorubicin [18]. Notch proteins are initially expressed as trans-membrane receptors at the plasma membrane. When activated by an extracellular ligand (for example Jagged or Delta), Notch1 undergoes a sequence of proteolytic cleavages, mediated by ADAM proteins and the γ-secretase complex, releasing an intra-cellular domain, Notch1^IC^. Notch^IC^ translocates to the nucleus and acts as a transcriptional co-regulator at a wide range of target genes [19]. Inhibitors that act at different stages of this activation cascade have been developed with a view to their potential use as therapeutics [20]. Of these, inhibitors of γ-secretase were developed first and are the most studied in terms of clinical trials for both Alzheimer’s disease [21, 22] and cancer [23], although they have proved to have problematic toxicity profiles in both contexts. The identification of new Notch inhibiting compounds with greater tolerability however promises further research into their use as anticancer agents in the future [24]. Here, we have explored the interplay between chemotherapy, Notch signalling, and expression and function of MRP1 in breast cancer, with a view to determining whether Notch inhibitors could play a role in reducing acquired chemotherapy resistance mediated by MRP1.

## Methods

### Patient selection and ethical approval

Ethical approval for use of patient samples and anonymised data was obtained from Leeds (East) Research Ethics Committee (06/Q1206/180) – written informed consent was taken from patients in accordance with this approval. The work complies with the Helsinki Declaration. Patients receiving NAC for primary breast cancer at LTH NHS Trust from 2005 to 2009 were identified. Cases to be studied further were selected to limit tumour heterogeneity. Inclusion criteria were: >3 years clinical follow-up after NAC; post-operative radiotherapy; grade 2 or 3 invasive ductal carcinoma on core biopsy; NAC regimen of anthracyclines +/- taxanes; matching pairs of biopsy and resection blocks available. Exclusion criteria were: inflammatory breast carcinoma; adjuvant chemotherapy in addition to NAC. Twenty-nine patients were identified for analysis. Patient and clinico-pathological characteristics were also collected (Table 1). Note that this cohort is a subset of a cohort analysed by us previously in relation to BCRP, Pgp and MRP1 expression [10].

**Table 1:** Clinico-pathological features of the patient cohort

Characteristic	Categories	No. of patients (%)
		*n* = 29
Age	<45	12 (41.4)
	>45	17 (58.6)
Grade (pre-NAC)	2	9 (31)
	3	20 (69)
Stage (pre-NAC)	T2	19 (65.5)
	T3	10 (34.5)
MRI response	Minimal	9 (31)
	Partial	20 (69)
NAC regimen	Epirubicin + cyclophosphamide (EC)	5 (17.2)
	EC + taxanes	24 (82.8)
Lymphovascular invasion	Positive	11 (37.9)
Axillary metastasis	Positive	15 (51.7)
Oestrogen receptor	Positive	17 (58.6)
Her2	Positive	3 (10.3)
Surgery	Breast conserving	11 (37.9 %)
	Mastectomy	18 (62.1 %)

### Immunohistochemistry

Immunohistochemistry was performed as described previously [10]. In summary, 5 μm sections were taken, and for each case matched biopsy and resection samples were placed on the same single slide (SuperFrost Plus; Menzel-Glaser, Braunschweig, Germany) to allow direct comparisons of staining between biopsy and resection. Slides were air-dried, and samples dewaxed with xylene and submerged in ethanol. Epitopes were retrieved by heat (900 W microwave, 10 min) in 10 mM citrate buffer (pH 6.0) and endogenous peroxidase activity was blocked using 0.3 % H_2_O_2_ (10 min). For anti-activated Notch1, non-specific primary antibody binding was blocked using casein (SP5020; Vector, Burlingame, USA) diluted 10-fold in antibody diluent reagent (Invitrogen, Carlsbad, USA) for 20 min. Slides were stained with the following primary antibodies in antibody diluent reagent for 1 h at room temperature; MRP1 (1:50, QCRL1 clone; sc18835, Santa Cruz Biotech, Santa Cruz, USA); activated Notch1 (Notch1^IC^: 1:100; ab8925, Abcam, Cambridge, UK). Both antibodies have been used previously for immunohistochemistry on breast tissue [25, 10], and for western blots with breast cell lines [26, 27]. Immuno-staining was visualised using Envision reagents (Dako, Gostrup, Denmark). Sections were counter-stained with Mayer’s haematoxylin and mounted in DPX (Fluka, Gillingham, UK). MRP1 expression was quantified using computer-aided scoring as a histoscore from 0 (no staining) to 300 (strong staining throughout epithelial areas) as described previously [10]. Notch1^IC^ nuclear staining was scored manually by two independent scorers (BK, BJW) using the Allred method [28, 29], giving scores from 0 (no staining) to 8 (strong staining in >66 % of tumour cells). Weighted Kappa coefficients (*k*) for the two independent scores were 0.78 for biopsies (good agreement), and 0.9 for resections (near perfect agreement); overall *k* = 0.89 (*n* = 58). Averages of the two scores were used as final expression scores.

### Cell Culture, transfection and drug treatments

Cell lines were obtained originally from the European Collection of Animal Cell Cultures. Cell line identities were confirmed (STR profiles, Leeds Genomics Service) and lines were consistently negative for mycoplasma (MycoAlert Mycoplasma detection assay, Lonza, Basal, Switzerland). Cells were maintained in DMEM supplemented with 10 % FCS (reagents from Invitrogen, Carlsbad, USA). Cells were seeded at either 1x10^5^ (HB2) or 2.5x10^5^ (other lines) cells/well in 6-well plates and left overnight before experiments unless otherwise stated. Trisilencer-27 siRNAs against Notch1-4 and scrambled controls were obtained from Origene (SR303207, SR303209, SR303210, SR303211, SR30004, Austin, USA). For transfection, cells were plated in 24-well plates and incubated overnight (to reach ~50 % confluence). Transfection was performed with 10nM siRNA with RNAiMAX (13778030, Invitrogen, Carlsbad, USA) in optimem for HB2 or Interferrin (409-10 Polyplus, Source Bioscience, Nottingham, UK) in DMEM for T47D according to the manufacturer’s protocols. Fresh medium was added after 6 h (HB2) or 24 h (T47D) and cells were harvested 72 h post-transfection. Drug stocks were stored in DMSO at -20 °C at 10 mM for doxorubicin hydrochloride (obtained from Leeds Hospital Teaching Trust), 1 mM for Calcein-AM (17783, Sigma, Poole, UK), or at 100 mM for DAPT (D5942, Sigma, Poole, UK) and MK571 (BMLRA1090005, Enzo Life Sciences, Exeter, UK). Vehicle control or drug was applied to cells for 24 h at 1 μM doxorubicin, 50 μM DAPT, 25 μM MK571 and 0.5 μM Calcein-AM unless otherwise stated.

### qPCR and western blots

For RNA analyses, mRNA was extracted using ReliaPrep with on-column DNase according to manufacturer’s instructions (Promega, Madison, USA). cDNA was synthesized using 500 ng RNA, GoScript and random primers (Promega, Madison, USA). Single RTs were performed for each sample and were assessed in duplicate wells by qPCR using SYBR green (Promega, Madison, USA) on an Applied Biosystems 7500 thermal cycler. Primers were designed using primer express v3 and sequences were (5’ to 3’): *hβACTIN*-F TTCTACAATGAGCTGCGTGTG, *hβACTIN*-R GGGGTGTTGAAGGTCTCAAA, *hABCC1*-F GGGACTCAGGAGCACACGAA, *hABCC1*-R AAATGCCCAGGGCTCCAT, *hHES1*-F AGGCGGACATTCTGGAAATG, *hHES1*-R CGGTACTTCCCCAGCACACTT, *hHEY1*-F GAAACTTGAGTTCGGCTCTAGG, *hHEY1*-R GCTTAGCAGATCCTTGCTCCAT, *hNOTCH1*-F GTCAACGCCGTAGATGACC, *hNOTCH1*-R TTGTTAGCCCCGTTCTTCAG. Quantification was performed using the ΔΔCT method [formula 2^-(treated cT target-gene – βactin)/(vehicle control cT target-gene – βactin)]. RT- and water only reactions were included and were essentially undetectable for all primer sets. For protein analyses, cells were lysed in RIPA buffer with protease inhibitor cocktail (Sigma, Poole, UK) and 10 mM PMSF. 25 μg protein was mixed with Laemmli buffer and heated (no heating for MRP1) before SDS-PAGE (4–15 % pre-cast polyacrylamide gels; Invitrogen, Carlsbad, USA). Samples were transferred to PVDF, blocked with 5 % milk/TBS-T and incubated with primary antibodies, anti-MRP1 (1/2000, MRPr1, ab83368, Abcam, Cambridge, UK) or anti-GAPDH (1/10,000, G9545, Sigma, Poole, UK) overnight (4 °C). Bound antibodies were detected with appropriate HRP conjugated secondary antibodies (1/2000, Dako, Gostrup, Denmark) and visualized with West-Pico or West-Femto diluted 1/5 with West-Pico (Pierce, Rockford, USA). Densitomtery was performed in ImageJ v1.47 and MRP1 values normalised to GAPDH and vehicle treated control.

### Enzyme-Linked ImmunoSorbent Assays (ELISAs)

Cells were plated in T25 flasks 24 h prior to treatments. Drugs were applied as described above and cells harvested after 24 h. Proteins were prepared and ELISA (Cleaved Notch1 cat. no. 7194; Cell Signalling, Beverly, USA) performed according to manufacturer’s protocols. Colorimetric readings were made at 450nM on an Opsis MR microplate reader (Dynex-Magellan, VA, USA) with Revelation quick-link software.

### Calcein-AM and doxorubicin retention assays

For Calcein-AM: 1x10^6^ cells were plated in T25 flasks and left overnight to adhere. Doxorubicin and DAPT were added at final concentrations of 1 μM and 50 μM respectively and left for 24 h. Where indicated, MK571 was added at 25 μM final concentration for 20 min. Cells were trypsinised, washed in PBS, suspended in complete RPMI and incubated in suspension in FACS tubes at 37 °C for 30 min. Cells were then loaded with Calcein-AM (0.2 μM final concentration) for 1 h at 37 °C. For washout, cells were washed in PBS three times and resuspended in complete RPMI. For analysis, cells were pelleted and resuspended in phenol-red free RPMI with 2 % FCS. 7AAD was added according to manufacturer’s recommendations. Flow cytometry for Calcein was performed using a LSRII Flow Cytometer (BD Bioscience, Franklin Lakes, USA) - 488 nm laser, 505 nm LP dichroic mirror and 530/30 nm bandpass filter with appropriate compensation to address any spillover from doxorubicin fluorescence (assessed using 550 nm LP dichroic mirror and 575/26 nm bandpass filter). For doxorubicin retention assays, cells were treated with either 50 μM DAPT, 25 μM MK571 or vehicle control for 24 h and then exposed to a repeat dose of DAPT, MK571 or vehicle control in the presence or absence of doxorubicin (1 μM) for 4 h. Cells were trypsinised and resuspended in phenol-red free RPMI with 1 % FCS. Flow cytomtery was performed using an Attune NxT Acoustic Focusing Imager (Life Technologies) and fluorescence was measured in the BL3 channel.

### Apoptosis assays

Cells were either treated with 1 μM DAPT (or vehicle control) for 24 h and then exposed to vehicle control, and a second DAPT treatment (1 μM) in the presence or absence of doxorubicin (10nM). Cells were trypsinised, washed in PBS and incubated with Annexin V-FITC and PI according to manufacturers recommendations (556547, BD Biosciences, Oxford, UK) for 1 h. Cells were analysed by flow cytometry using a LSRII Flow Cytometer (BD Bioscience, Franklin Lakes, USA). Cells positive for Annexin V, PI or both were classified as apoptotic.

## Results

### MRP1 and Notch1^IC^ are up-regulated in breast cancers by chemotherapy

**Fig. 1:**
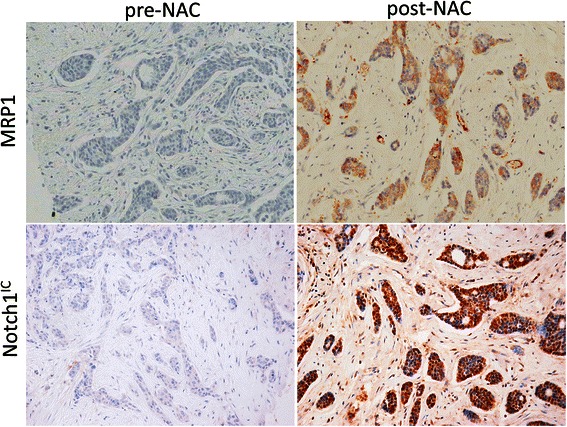
Representative staining patterns of MRP1 and Notch1^IC^ in matched breast tumour tissues pre- and post-NAC. Immunohistochemistry was performed on matched tissues from pre-NAC diagnostic core biopsies (left) and post-NAC surgical resection samples (right) for either MRP1 or activated Notch1 as labelled

**Fig. 2:**
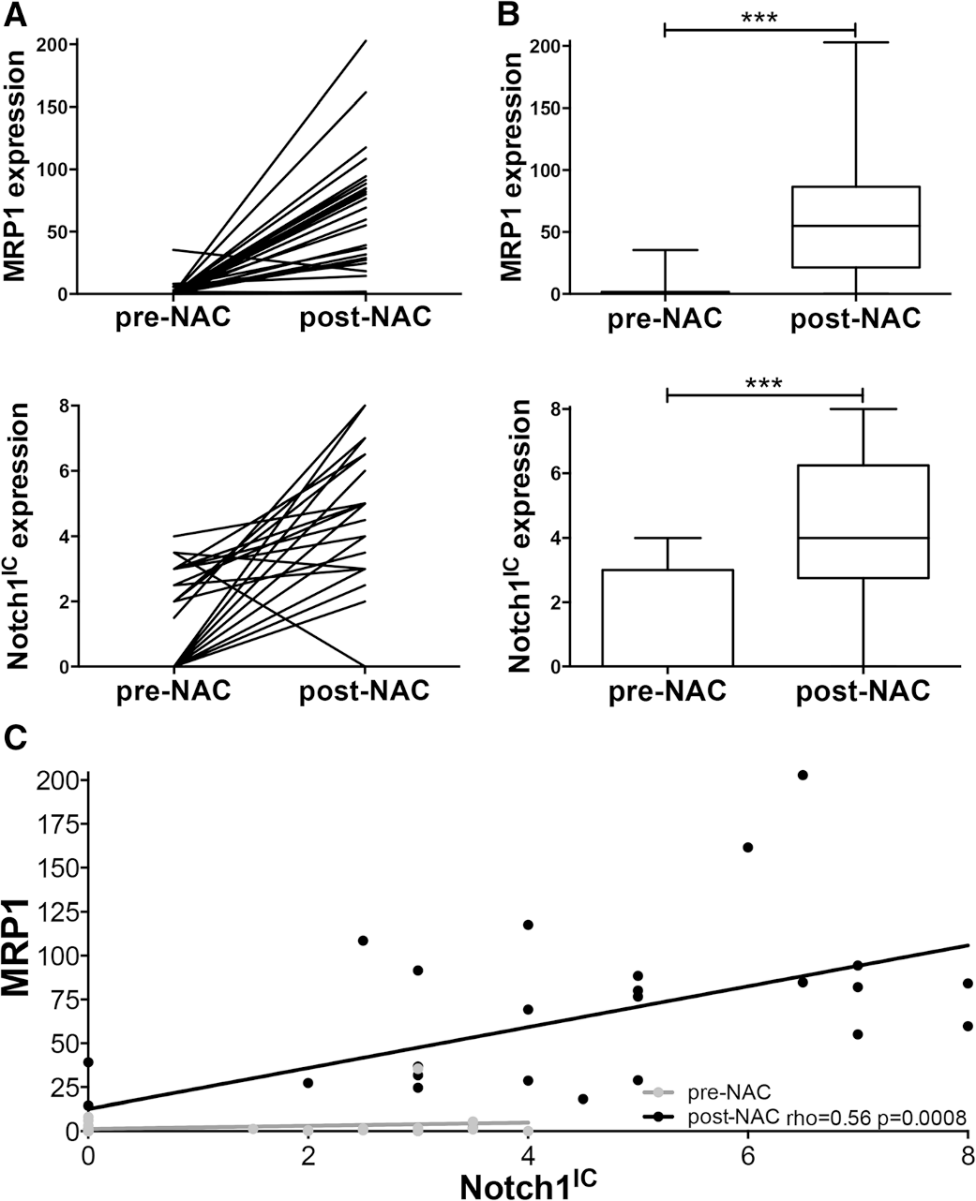
MRP1 and Notch1^IC^ are induced by NAC in breast cancer patients and their expression is significantly positively associated post-NAC. Immunohistochemistry for either MRP1 or activated Notch1 was performed and quantified on matched pre- and post-NAC breast cancer tissues from 29 patients. **a** Expression of MRP1 (top) and Notch1^IC^ (bottom) is shown for each individual patient (each black line) both pre- and post-NAC. **b** Expression of MRP1 and Notch1^IC^ across the cohort as box and whisker plots showing median (line), 25^th^-75^th^ percentile (box) and min to max values (whiskers). Wilcoxon ranked score, *p* < 0.0001 for both MRP1 and Notch1^IC^. **c** A scatter plot of MRP1 and Notch1^IC^ expression with post-NAC demonstrating a significant positive correlation (rho coefficient 0.5884, *p* = 0.0008)

In order to assess the relevance of MRP1 expression and Notch signalling in chemotherapy treatment, the expression levels of MRP1 and activated Notch1, Notch1^IC^, were examined using immunohistochemistry in tumours from 29 breast patients treated with NAC. Clinical and pathological features of this cohort are shown in Table 1. Expression was examined both pre-NAC, using diagnostic biopsies, and post-NAC, using matched surgical resections. Representative staining patterns for each antigen are shown (Fig. 1) and staining was quantified (Fig. 2). MRP1 and Notch1^IC^ were absent or expressed at very low levels prior to NAC, while positive expression of both was typical post-NAC, although with considerable variation in levels between tumours. Expression of MRP1 or Notch1^IC^ did not significantly correlate with any of the clinical or pathological features listed in Table 1 (although it should be noted that this analysis is limited by the relatively small cohort size). However, both MRP1 and Notch^IC^ were significantly up-regulated after NAC (Wilcoxon signed-rank test *p* < 0.0001; Fig. 2b), and their expressions were significantly positively associated post-NAC (rho coefficient 0.6, *p* = 0.0008), but not prior to treatment (Fig. 2c). We concluded that our data were compatible with Notch1 signalling playing a role in chemotherapy-induced up-regulation of MRP1 expression.

### Doxorubicin induces Notch targets and MRP1 in breast cell lines

**Fig. 3:**
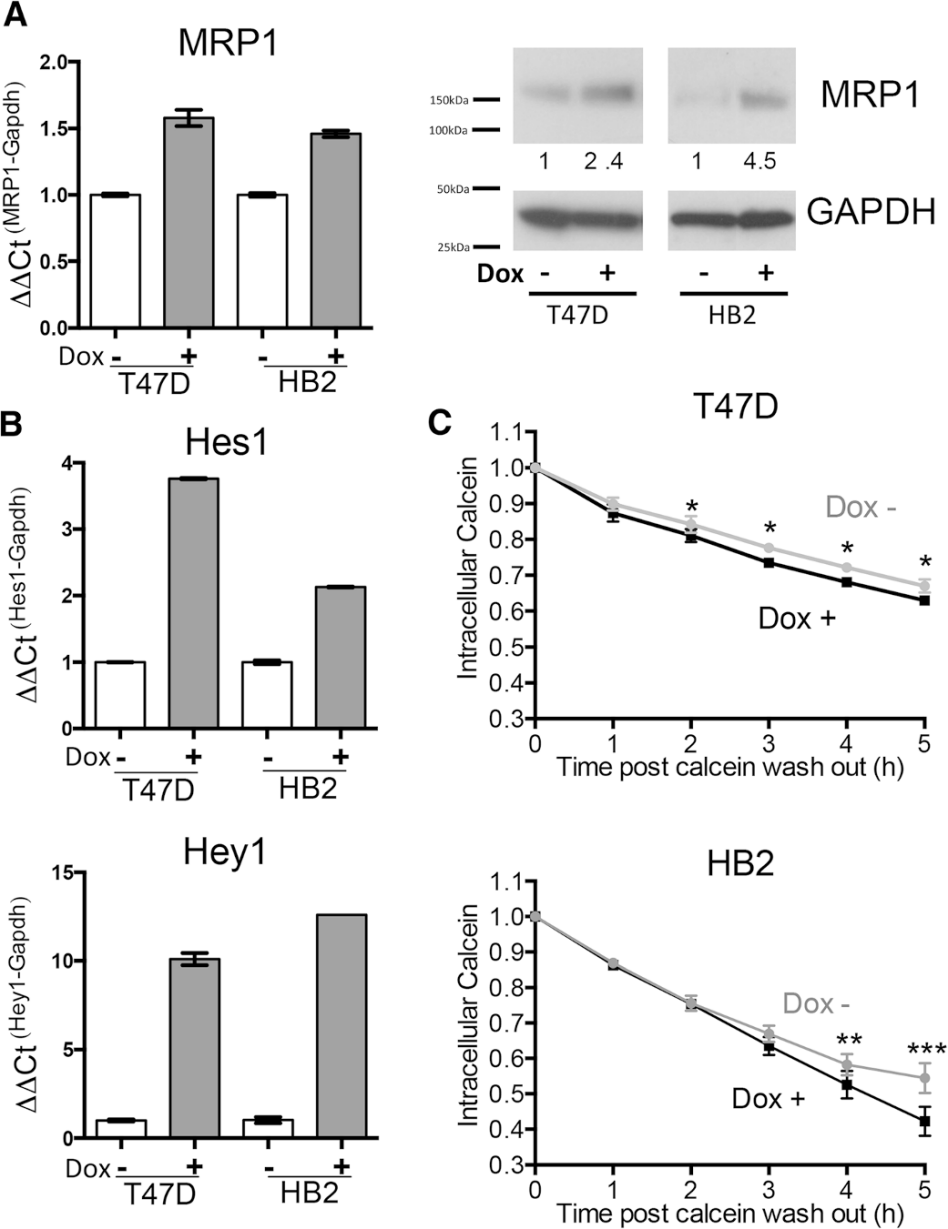
MRP1 expression and function are induced in breast cell lines by doxorubicin *in vitro*. HB2 or T47D cells were treated for 24 h with 1 μM doxorubicin or vehicle control (DMSO). **a** MRP1 expression was quantified by qPCR (left) or Western blot (right). For qPCR, means with SD of triplicate PCR reactions are presented. Densitometry values are presented beneath MRP1 blots and pertain to the blots presented. Data for qPCR and Western blot are representative of at least 2 independent biological replicates. **b** Expression of canonical Notch target genes Hes1 and Hey1 was quantified by qPCR. Means are presented with SD of triplicate PCR reactions, and experiments are representative of at least 2 biological repeats. **c** Efflux of the fluorescent MRP1-substrate, esterified calcein, was assessed using flow-cytometry. Efflux was significantly enhanced in the presence of doxorubicin (non-linear regression T47D *p* = 0.0035, HB2 *p* = 0.0009). Analysis of individual timepoints reveal T47D cells show significantly enhanced efflux over 2-5 h post-wash out (two-tailed *t*-test *p* < 0.05 at each time point), whilst significant efflux occurred from 4 to 5 h post-wash out for HB2 cells (two-tailed *t*-test *p* < 0.01 at 4 h and *p* < 0.0001 at 5 h). The mean response of 4 independent biological replicates with technical triplicates is presented. Error bars represent the SEM of the 4 replicates

To determine whether Notch-dependent regulation of MRP1 could be demonstrated *in vitro*, two breast cell lines were used: the ER positive cancer line T47D alongside the non-malignant but immortalised HB2 line. Cell lines were treated with the anthracycline doxorubicin, or with vehicle control, for 24 h and MRP1 expression was examined by qPCR and western blotting (Fig. 3a). MRP1 expression was increased after doxorubicin addition in both cell types. In addition, expression of the well-established Notch target genes Hes1 and Hey1 were found to be induced after exposure to doxorubicin (Fig. 3b), consistent with activation of Notch signalling. MRP1, Hes1 and Hey1 expression were all also increased after doxorubicin treatment in the MCF7 breast cancer cell line (Additional file 1: Figure S1). MRP1 activity was assessed by quantification of the export of the fluorescent MRP1 substrate esterified calcein using flow-cytometry. T47D or HB2 cells were treated with doxorubicin or control as before, and were then loaded with the dye. Intracellular levels of esterified calcein were assessed hourly after the dye was removed from the medium (Fig. 3c). In both cell lines, doxorubicin treated cells exported calcein significantly more efficiently than the controls (non-linear regression: T47D *p* = 0.0035; HB2 *p* = 0.0009), although these differences were relatively small. We concluded that doxorubicin induced MRP1 expression and activity in both cell lines.

### Doxorubicin induces Notch1 activation and Notch-dependent activation of MRP1 in T47Ds but not HB2s

**Fig. 4:**
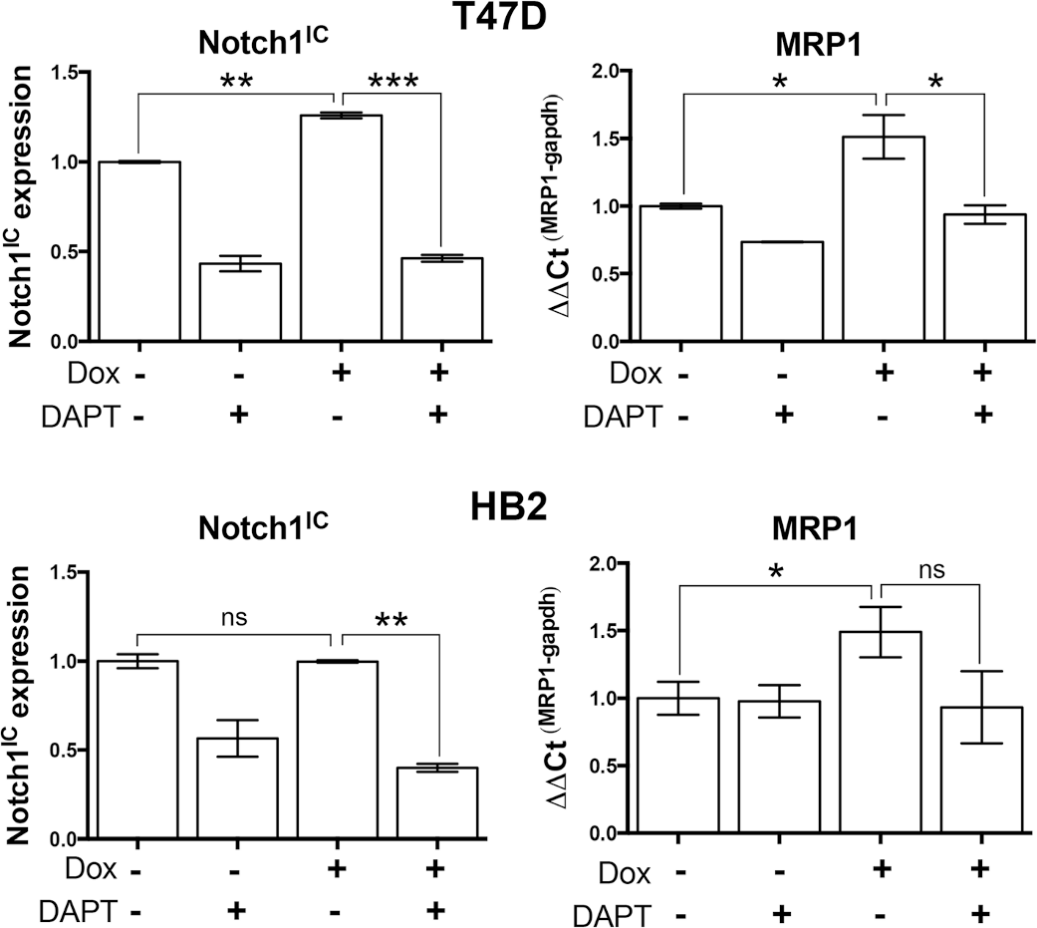
Doxorubicin-induced expression of MRP1 in T47D cells is abrogated by inhibition of Notch1 activation. Cells were treated for 24 h with control, 1 μM doxorubicin, 50 μM DAPT (an inhibitor of Notch function) or both in combination. Left: Expression Notch1^IC^ of was determined using ELISAs. Means with SEM of two independent biological replicates are presented. Right: MRP1 mRNA expression was quantified using qPCR. Means with SEM of two independent biological replicates are presented. T-tests were used to determine significant changes. * *p* < 0.05, ** <0.01, *** < 0.001, ns = not significant

Next, we aimed to determine whether this MRP1 up-regulation was susceptible to therapeutic intervention by inhibiting Notch function. The γ-secretase inhibitor DAPT was used to prevent cleavage and functional activation of Notch [30]. T47D or HB2 cells were treated with vehicle control, DAPT, doxorubicin or a combination of doxorubicin and DAPT. Notch1 activity was assessed directly using ELISAs for Notch1^IC^ while MRP1 expression was assessed using qPCR (Fig. 4). In T47D cells, DAPT reduced basal Notch1 activity as expected, while doxorubicin induced Notch1 activity. In the combination treatment, DAPT completely inhibited the doxorubicin-induced increase in Notch1 activity (top left panel). This was associated with similar changes in MRP1 expression (top right panel): DAPT reduced basal MRP1 levels and, critically, blocked doxorubicin-induced MRP1 expression. These data support the conclusion that doxorubicin drives a Notch1-dependent increase in MRP1 expression and that this increase can be inhibited by DAPT. The result was less clear-cut in HB2 cells (bottom panels). DAPT reduced Notch1 activity as expected, but there was no evidence for doxorubicin inducing Notch1 activity in this cell line, despite the previous observation of Hey1/Hes1 induction (Fig. 3b). Furthermore, DAPT had no impact on basal MRP1 expression. However, doxorubicin did induce MRP1 expression, and this induction demonstrated a trend to be reduced by DAPT (not significant). These data support Notch1-independent actions on MRP1 expression by doxorubicin, as MRP1 expression increased in the absence of an increase in Notch1 activity.

**Fig. 5:**
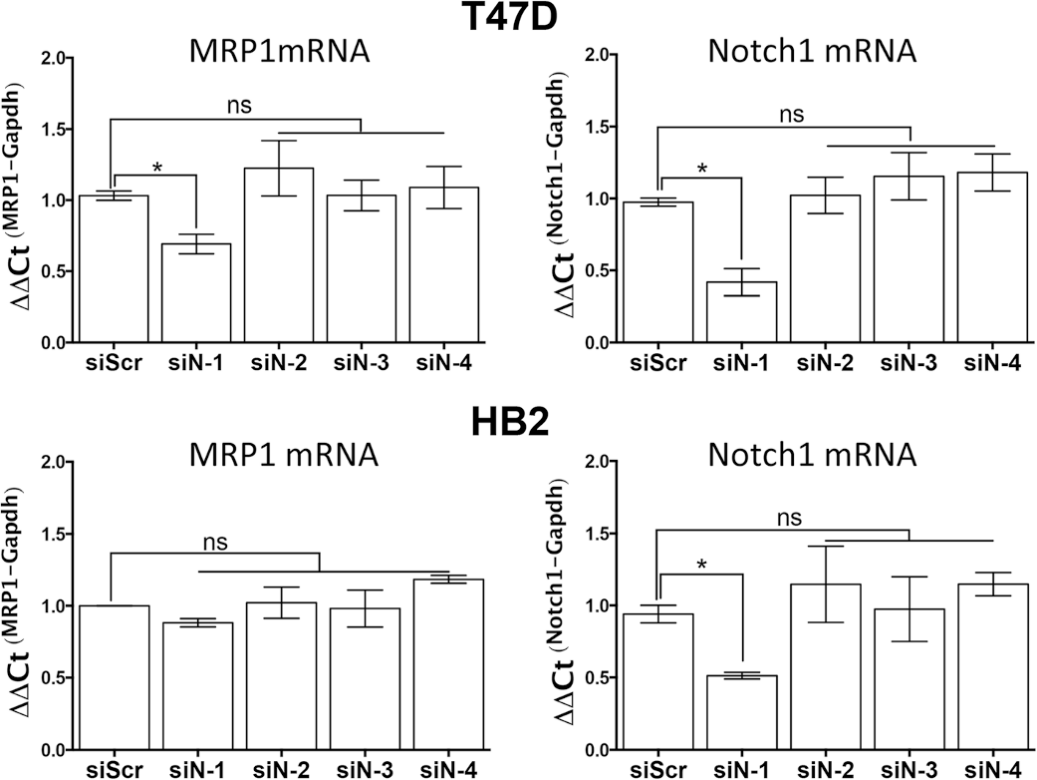
siRNA knock-down of Notch1 reduces basal expression of MRP1 in T47Ds but not HB2s. Cells were transiently transfected with control or Notch1, 2, 3 or 4-targetted siRNAs for 72 h. Expression of Notch1 andABCC1/MRP1 was quantified using qPCR and is presented relative to siScrambled (control) transfected levels. Mean levels from 3 independent siRNAs to each target are presented and results show mean of 2 biological repeats (error bars represent SEM). T-tests were used to determine significant changes, * *p* < 0.05, ns = not significant

With the potential role of other γ-secretase targets in mind, we then sought to confirm the role of Notch1 in controlling MRP1 levels using siRNA approaches. T47D or HB2 cells were transfected with siRNAs against each of Notch1-4, and we examined whether this reduction in basal activity of these Notch molecules would impact on MRP1 expression using qPCR (Fig. 5). Targeting of Notch1, but not the other Notch molecules, reduced MRP1 expression in T47D cells (left top panel), but not in HB2 cells (left bottom panel). As a control, we also confirmed that Notch1 siRNAs had indeed successfully reduced Notch1 expression in both cell lines (right panels). These data further support the conclusion that MRP1 expression is dependent on Notch1 in T47D cells but not in HB2 cells.

### Inhibition of Notch activation reduces doxorubicin-induced MRP1 activity in T47Ds but not HB2s

**Fig. 6:**
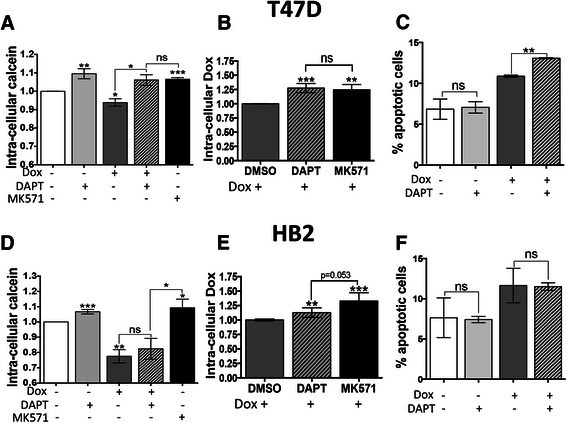
Inhibition of Notch activation reduces doxorubicin-induced MRP1 function and, in T47D cells, enhances doxorubicin-dependent apoptosis. **a** Intracellular calcein levels in T47D cells were quantified 5 h after calcein wash-out following 24 h treatment with control, DAPT (50 μM), doxorubicin (1 μM), doxorubicin and DAPT, or 0.5 h treatment with the ABC pump-inhibitor MK-571 (25 μM). Data are presented as mean with SEM of 2 biological replicates. **b** Intra-cellular doxorubicin levels in T47D cells were quantified after treatment with either doxorubicin alone (1 μM for 4 h), or in combination with DAPT (pre-treated with 50 μM for 24 h) or MK-571 (pre-treated with 25 μM for 20 min). Data are presented as mean with SEM of 2 biological replicates. **c** Apoptosis was measured in T47D cells after doxorubicin (10nM for 24 h) and DAPT (1 μM) treatment, alone and in combination. Data are presented as mean with SEM of 2–4 biological replicates per bar. Asterisks represent significant changes compared to vehicle control or between test conditions as indicated (two tailed *t*-test * *p* < 0.05, ** *p* < 0.01, *** *p* < 0.001, ns = not significant). **d**-**f** as described for A-C but in HB2 cells

Next, we assessed whether the changes in MRP1 expression associated with doxorubicin and/or DAPT treatment are reflected in changes in export activity and subsequent cell survival. MRP1 substrate-export activity was tested using the calcein loading assay as previously, and in addition we used the natural fluorescence of doxorubicin as a further measure of cellular export potential. For calcein assays, we present the relative amounts of fluorescent dye remaining in cells after 5 hours wash-out of calcein-AM from the medium - greater MRP1 activity leads to more dye export and lower levels of cellular fluorescence (Fig. 6a and d). In doxorubicin assays, efflux potential is presented as relative amount of intra-cellular doxorubicin (Fig. 6b and e). For both assays, we have included a further control, MK571, which is a chemical inhibitor of MRP1 function and represents the maximal increases in fluorescence that can be achieved by functional inhibition of MRP1.

In T47D cells, treatment with DAPT significantly increased calcein retention (reflecting reduced MRP1 activity), while treatment with doxorubicin reduced calcein retention (reflecting increased MRP1 activity) (Fig. 6a). Critically, in the combination treatment DAPT abolished the doxorubicin-induced export of calcein, indeed calcein was retained to the same degree as after chemical inhibition of MRP1 using MK571, demonstrating that DAPT was highly-effective at inhibiting MRP1-dependent export in this context. A similar result was seen when fluorescence from doxorubicin itself was assessed (Fig. 6b) – both DAPT and MK571 induced doxorubicin retention, again reflecting reduced MRP1 function. We concluded that DAPT reduces doxorubicin-induced export, thereby increasing doxorubicin-loading of the cells. To test the phenotypic effect of this conclusion, Annexin-V/PI assays were performed to assess apoptosis after the various treatments (Fig. 6c). DAPT had no effect on apoptosis alone, while doxorubicin – as expected for a chemotherapeutic agent – induced apoptosis. The combination of DAPT with doxorubicin caused a larger increase in apoptosis (Fig. 6c, *t*-test *p* = 0.0012) indicating that the DAPT-induced increase in doxorubicin-loading seen in Fig. 6b was cytotoxic. These data support the use of the combination treatment as a potential therapy.

As before, the result in HB2 cells was less clear. Doxorubicin treatment greatly reduced calcein retention and this was not significantly altered by addition of DAPT (Fig. 6d) – a result that is compatible with our conclusion from Figs. 4 to 5 that doxorubicin-induced increased expression of MRP1 is likely to be independent of Notch1 signaling in this cell line. However, more surprisingly, treatment with DAPT alone significantly increased calcein retention (Fig. 6d), a finding we can not explain in terms of MRP1 activity since we previously showed DAPT not to reduce basal MRP1 expression (see Fig. 4). When using doxorubicin fluorescence as the marker of export, DAPT caused only a very slight increase in doxorubicin-loading (Fig. 6e). Finally, DAPT had no influence on apoptosis either with or without doxorubicin (Fig. 6f). In HB2 cells, these data demonstrate that DAPT has some influences on export activity that are independent of MRP1, and that the combination of doxorubicin and DAPT does not cause synergistic cell killing.

## Discussion

MRP1 expression has been studied extensively as a potential predictor of chemotherapy response and/or clinical outcome in a wide range of cancers [4, 9, 31, 32]. Some studies demonstrated that high MRP1 expression was associated with chemotherapy resistance and poor survival, presumably on account of the ability of MRP1 to export chemotherapeutics from the tumour cells thereby enhancing their survival. However, previous work from our laboratory reported that MRP1 was expressed only at very low levels in breast cancers prior to chemotherapy and therefore provided few prognostic or predictive insights [10]. Interestingly, MRP1 expression in breast tumours was strongly induced by NAC, specifically implicating up-regulated MRP1 as a response to therapy and a potential mediator of the development of acquired resistance. By identifying and therapeutically targeting the mechanisms that allow up-regulation of MRP1 it might be possible to selectively sensitize cancer cells to chemotherapy.

Two published studies, discussed in detail below, allowed development of the hypothesis that activity of Notch1 mediates MRP1 induction in some breast cancers. First, Gonzalez-Angulo *et al.* compared gene expression profiles of breast cancers before and after NAC and used *in silico* analyses of differences to identify signalling pathways influenced by NAC [13]. In the non-basal subset of tumours, Notch signalling was the most significantly up-regulated pathway identified, although it should be noted that this prediction was based solely on bioinformatics and was not subject to any validation. Secondly, Cho *et al.* identified MRP1 as a direct transcriptional target of Notch1 signalling in an etoposide-resistant variant of the MCF7 breast cancer cell line, and identified the specific promoter region allowing Notch1-dependent MRP1 regulation [12]. This regulation was not confirmed in a clinical setting or related to chemotherapy in this original paper, although recent support comes from work showing Notch1-dependent up-regulation of MRP1 to increase chemo-resistance in stem-like cells from prostate cancer lines [33]. The data presented herein are based on direct measures of activated Notch1 and show that Notch signalling is indeed induced by chemotherapy in both clinical breast cancer (Fig. 2) and breast cell lines (Figs. 3 and 4), confirming the previous bioinformatic prediction of Gonzalez-Angulo *et al*. Furthermore, these data show that activation of Notch1 at the protein level correlates significantly with induction of MRP1 expression (Fig. 2c) and crucially, confirm clinically the cell line-based findings of Cho *et al*. Further clarification of this mechanism was made by the observation that only Notch1, as opposed to Notch2-4, regulates MRP1 expression (Fig. 5).

**Fig. 7:**
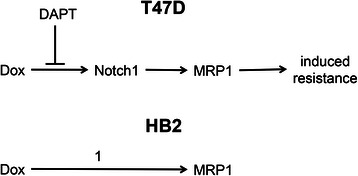
Schematic representing the differences between T47D and HB2 cells following exposure to doxorubicin. Activation of MRP1 and induction of chemoresistance in T47D cancer cells is Notch1 dependent and can be inhibited by DAPT, representing a potential chemo-sensitizing strategy. In HB2 cells, doxorubicin induces MRP1 expression but this is independent of Notch1 signalling (1), therefore Notch inhibitors do not impact on chemo-sensitivity

Most importantly, this study investigated whether chemotherapy-induced MRP1 regulation can be inhibited with potential therapeutics, using a luminal breast cancer cell line (in accordance with the bias noted by Gonzalez-Angulo *et al.* to non-basal cancers) and an immortalised non-cancer breast epithelial line. Notch regulation of MRP1 has not been investigated in non-tumouriogenic cells previously. The well-characterised γ-secretase inhibitor DAPT was used as an inhibitor of Notch1 activation. γ-secretase inhibitors have a long history of use in clinical trials for Alzheimer’s disease [34] and more recently cancer [35–37] including in breast [38], and their use in combination with chemotherapy has been proposed previously [39]. Our results demonstrate differences between the two breast cell lines tested (see Fig. 7 for a flow-scheme). First, MRP1 and canonical Notch-targets were induced by doxorubicin in both cell lines (Fig. 3). However, Notch1 was only activated by this treatment in the T47D cancer cells and not in the non-cancer HB2 cells (Fig. 4), and MRP1 induction was dependent on Notch1 activity only in the cancer cells (Figs. 4 and 5). These differences were reflected by functional influences of MRP1 both directly at the level of export of substrates and at the level of induction of apoptosis by doxorubicin (Fig. 6). In T47D cells, inhibition of Notch activation caused an increased loading of doxorubicin and enhanced cell killing, while in the non-cancer HB2 cells this treatment had minimal effects on doxorubicin loading and no significant effect on cell killing. It is interesting to note that the combination treatment enhanced chemotherapy-efficacy in the cancer cell line, but not in the non-cancer line indicating a potential degree of cancer-specificity that might allow synergistic killing of the cancer cells while sparing normal cells, although clearly this remains speculative until confirmed in further appropriate models.

## Conclusions

We propose that inhibition of Notch signalling may enhance the efficacy of chemotherapy for breast cancer and support the use of Notch inhibitors in clinical trials testing this hypothesis.

## Additional file

Additional file 1: Figure S1. MRP1 expression is induced in MCF7 cells by doxorubicin in vitro. MCF7 cells were treated for 24 h with 1 μM doxorubicin or vehicle control (DMSO). **A)** MRP1 expression was quantified by qPCR (left) or Western blot (right). For qPCR, means with SD of triplicate PCR reactions are presented. Densitometry values are presented beneath MRP1 blots and pertain to the blots presented. Data for qPCR and Western blot are representative of at least 2 independent biological replicates. **B)** Expression of canonical Notch target genes Hes1 and Hey1 was quantified by qPCR. Means are presented with SD of triplicate PCR reactions, and experiments are representative of at least 2 biological repeats. (TIFF 216 kb)

## Abbreviations

ABC: ATP-binding cassette
DAPT: N-[N-(3,5-Difluorophenacetyl)-L-alanyl]-S-phenylglycine t-butyl ester
ELISA: Enzyme-Linked ImmunoSorbent Assay
MRP1: Multi-drug Resistance associated Protein-1
NAC: neoadjuvant chemotherapy
Notch1^IC^: Notch1 intracellular domain
PI: propidium iodine
qPCR: quantitative polymerase chain reaction
