# Supplementary figures and images for: Chemotherapy induces Notch1-dependent MRP1 up-regulation, inhibition of which sensitizes breast cancer cells to chemotherapy
Source: BMC Cancer. 2015 Sep 11;15:634. doi: 10.1186/s12885-015-1625-y (PMC4567818; doi:10.1186/s12885-015-1625-y)

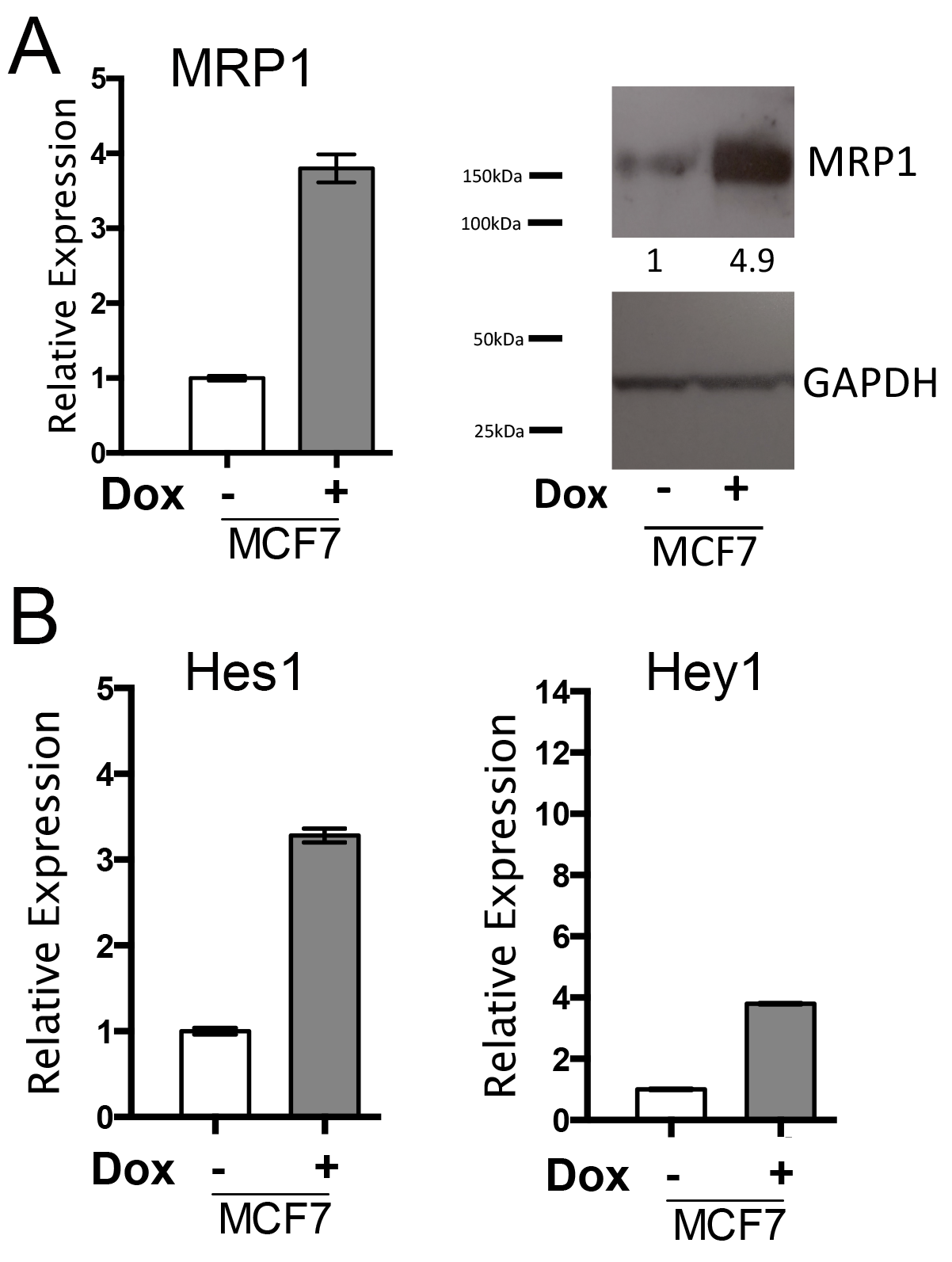

Supplement: Additional file 1: Figure S1. — MRP1 expression is induced in MCF7 cells by doxorubicin in vitro. MCF7 cells were treated for 24 h with 1 μM doxorubicin or vehicle control (DMSO). A) MRP1 expression was quantified by qPCR (left) or Western blot (right). For qPCR, means with SD of triplicate PCR reactions are presented. Densitometry values are presented beneath MRP1 blots and pertain to the blots presented. Data for qPCR and Western blot are representative of at least 2 independent biological replicates. B) Expression of canonical Notch target genes Hes1 and Hey1 was quantified by qPCR. Means are presented with SD of triplicate PCR reactions, and experiments are representative of at least 2 biological repeats. (TIFF 216 kb) [file 12885_2015_1625_MOESM1_ESM.tiff]
